# Stakeholder dialogue on dilemmas at work as a workplace health promotion intervention including employees with a low SEP: a Responsive Evaluation

**DOI:** 10.1186/s12889-022-12802-z

**Published:** 2022-02-28

**Authors:** Hanneke van Heijster, Jantien van Berkel, Cécile R. L. Boot, Tineke Abma, Emely de Vet

**Affiliations:** 1 Department of Social Sciences, Chair Group Consumption & Healthy Lifestyles, Wageningen University & Research, Hollandseweg 1, 6706 KN Wageningen, Netherlands; 2 Department of Public and Occupational Health, Amsterdam Public Health Research Institute, Amsterdam UMC, VU University, Amsterdam, Netherlands; 3 Department Public Health, Leiden University Medical Centre, Leiden University, Leiden, Netherlands

**Keywords:** Workers, Blue-collar, Health promotion, Worksite, Intervention, Participation, Participatory, Co-creation

## Abstract

**Background:**

The aim of this study was to evaluate the perceived changes of an innovative workplace health promotion intervention and evaluation. In this study, a bottom-up approach was taken to define the central themes and relevant outcomes of an intervention. These central themes and relevant outcomes of the intervention were defined together with stakeholders, including employees with a low socioeconomic position.

**Methods:**

The intervention consisted of a series of structured stakeholder dialogues in which dilemmas around the – by employees defined —health themes were discussed. The intervention was implemented in a harbor service provider with approximately 400 employees. Over a two-year period, 57 participants engaged in eight dialogues of one hour. 15 interviews and six participant observations took place for the evaluation of the intervention.

**Results:**

Together with the stakeholders, high workload and mental health were defined as central themes for the dialogue intervention in the male-dominated workplace. The dialogue intervention contributed to changes, on different levels: individual, team, and organization. Overall, the stakeholder dialogues advanced the understanding of factors contributing to high workload and mental health. In reply to this, several actions were taken on a organizational level.

**Conclusions:**

Taking a bottom-up approach in WHP allows to understand the health issues that are important in the daily reality of employees with a low socioeconomic position. Through this understanding, workplace health promotion can become more suitable and relevant for employees with a low socioeconomic position.

**Trial registration:**

Netherlands Trial Register (NRT): NL8051. Registration date: 28/09/2019, Retrospectively registered https://www.trialregister.nl

**Supplementary Information:**

The online version contains supplementary material available at 10.1186/s12889-022-12802-z.

## Background

Significant health inequalities between individuals with low a socioeconomic position (SEP) and a high SEP exist in most Western-European countries [1, 2]. Life expectancy of individuals with a low SEP can be up to 10 years shorter than of individuals with a high SEP [3]. Also, individuals with a low SEP live between 10 to 23 years shorter in good health [4]. Workplace health promotion (WHP) is considered promising to improve health of employees with a low SEP. The workplace gives access to part of the generally hard to reach low SEP population, as half is employed [5] and employees spend much time of their lives at work [6, 7]. Also, the workplace offers a physical and social infrastructure necessary for health promotion [8]. Therefore, WHP has the potential to contribute to the reduction of health inequalities.

However, it is doubtful if WHP in its current form does contribute to the reduction of health inequalities. Recent Individual Participant Data (IPD) meta-analyses on in total 15 Dutch WHP intervention studies, showed no effects on BMI [9]—except from small effects for high-risk groups under specific conditions—and no effects on lifestyle behaviors [10] of employees with both low and high SEP, and no effects on self-perceived health of employees with a low SEP [11]. A meta-analysis including mainly intervention studies from the US found some evidence that physical activity interventions at work may be effective in reducing health inequalities, but the evidence base was small and of low quality [12].

Three possible underlying reasons for the disappointing effects of current WHP have been described before [13]. First, the lack of acknowledgement of diverging values and interests of the many stakeholders involved in WHP, such as employers, employees, intervention providers, research and knowledge institutes and insurance companies [14]. These perspectives may often be competing, possibly affecting the effects and relevance of WHP [15]. Second, WHP evokes ethical questions. For example about who is responsible for employees’ health and what this responsibility entails [16], whether and to which extent interference in privacy of employees is acceptable, and about voluntariness of participation while power dependencies between employer and employee in the workplace exist [14, 16, 17]. Third, employees with a low SEP generally lack voice in the design and evaluation of WHP [14], being rather researched upon, than with [15]. Involving employees in WHP—those with first-hand experience of the particular workplace—may increase its relevance [15]. This first-hand experience is especially relevant when it comes to employees with a low SEP, as insight in how to target their health effectively considering their lifeworld, is scarce [18]. WHP may be more suitable when deliberate attention is paid to the aforementioned underlying reasons.

This study involves an innovative WHP intervention and evaluation in which the underlying reasons for previous limited effects are taken into account. A bottom-up approach is taken to define the central themes for the intervention, where special attention is paid to involve employees with a low SEP through a participatory approach to evaluation: Responsive Evaluation [19]. In Responsive Evaluation stakeholders are active partners in defining central themes and relevant research changes [20]. To date, it has been more common in WHP that central themes and outcomes of an intervention are defined by the researchers [14]. Also, being involved in defining central themes may enhance the relevance of WHP for employees with a low SEP [20], thereby offering a possible solution for low participation of employees with a low SEP [21–23].

The intervention consists of a series of structured stakeholder dialogues, in which participants discuss dilemmas around the central health themes. Participants bring in experiences from their daily experiences [13]. Rather than an educative or counseling component, the experiences of participants are central in the intervention. By bringing together and confronting a variety of perspectives in the dialogues, a learning process can emerge and shared insights can be gained. This learning process can take place at various levels, including the case, individual, team and organizational level [24].

The aim of this study is to evaluate stakeholder dialogue as an intervention for WHP in two ways. First, together with stakeholders, themes for and the desired outcomes of the dialogues will be defined. Second, it will be evaluated with stakeholders whether and which changes are perceived during and after the stakeholder dialogue.

## Methods

An extensive description of methods was provided in the Study Protocol of this study published elsewhere [13].

### Setting

During two years, the study was conducted in a harbor service provider (industrial sector) with approximately 400 employees, in The Netherlands.

### Design

**Fig. 1:**
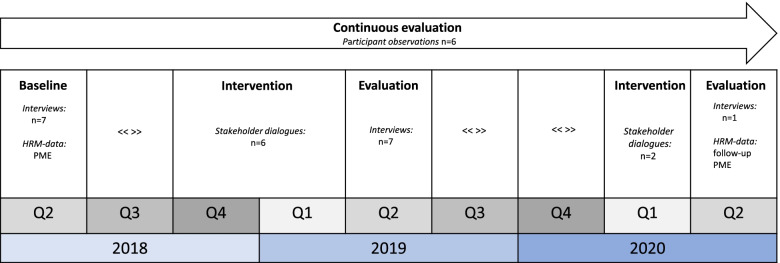
Schematic overview of the Responsive Evaluation and Intervention [PME = Periodic Medical Evaluation. <  <  >  > indicates that there was no research activity during this period]

The intervention was evaluated through Responsive Evaluation, a participatory form of evaluation [20]. Responsive evaluation constitutes a iterative research process in which data collection and analysis partly overlap [25]. More details about this form of evaluation are described elsewhere [13]. Methods in the two-year evaluation were interviews, participant observations and HRM-data (Fig. 1). These methods were used for two purposes. First, to define the themes and relevant outcomes for the stakeholder dialogues. Second, to evaluate changes after or during the stakeholder dialogues, as perceived by the stakeholders.

### Intervention

The aim was to perform five to six stakeholder dialogues per year [13]. Base for the stakeholder dialogues was a form of moral case deliberation (MCD), namely the Dilemma-method [26]. In short, in a dialogue according to the Dilemma-method, participants bring in dilemmas they face in their daily work (e.g. should I as for help if I’m too busy, while I think this is not accepted in the culture of this organization?). The dialogue facilitator helps participants to look at the different perspectives (interests, values, norms) on this dilemma, for example the perspective of the employee, employer, client and colleagues. After evaluating the different options for action in this dilemma, all participants formulate what they would to in the situation. Differences between solutions are discussed according to the rules of dialogue (e.g. postpone judgments). Also, the participants deliberate about the individual and/or organizational actions that are necessary to act in the desired manner. The emphasis on mutual learning among participants and the focus on the ethical dimensions of issues and experiences of participants differs MCD from similar methods such as focus groups and health circles [27]. This form was considered best suitable for the purpose of the project [13].

The Dilemma-method was adapted in various ways to make it suitable for the work setting and its employees [13]. To date, the Dilemma-method has not been used specifically for workplace health promotion and with employees with a low SEP. The method is traditionally used for health care professionals to deliberate about dilemmas they encounter in their daily care-practices [28]. Several adaptations were made to make the dialogue method feasible for the setting of this study. These adaptations are explained in Additional file 1, which includes the dialogue guide and also describes the adaptations made throughout the evaluation based on advancing insights.

### Sampling and recruitment

A proportionate universalism approach was taken to recruit participants [29]. This means that all stakeholders were eligible to participate. However, special attention was paid to include employees with a low SEP, as they generally participate less in WHP interventions. Employees with a higher SEP were not excluded from the intervention, because the intervention was based on the rationale that context changes would benefit employees with low SEP the most. In other words, the entire organisation was eligible for participation as they constitute the (social) context.

Stakeholders could participate in the intervention (stakeholder dialogues) and evaluation (interviews, participant observations), but also to one of both. Participants were recruited for the intervention and evaluation via contact persons in the organization. Participation was based on willingness to participate. Operational employees were asked to indicate with whom they would feel comfortable enough to have the dialogues with, as a prerequisite for a safe communication climate. Mixed groups with employees and direct supervisors from different departments were preferred.

Participants received an email from the researchers (HvH, JvB) with information about the dialogue (duration, location, aim) and explanation for preparation. Participants were invited to think of a dilemma related to the central themes, that were defined earlier in the Responsive Evaluation. The aim was to have six to twelve participants per dialogue.

### Participants

In total there were 16 participants in 15 interviews. Participants worked at various departments in the organization (management or support staff (7), supervisors (4), and operational employees (5)). Participant observations were performed at two operational departments during three toolbox sessions, that were attended by operational employees. In the dialogues, 57 participants participated over eight dialogues. The number of participants in the dialogues ranged from four to 11. The majority was male (90%). 20.5% of all participants were operational staff with a low SEP (low educational requirement). *Educational requirement* was defined by the researchers and was based on the educational level required for the job. Participants were not asked for their educational level to avoid stigmatization. Educational requirement was used as an estimation. The group composition of the dialogues was determined based on the preferences of operational employees [25].

In the first year, six dialogues took place, and two in the second year. The lower number of dialogues in the second year was the result of 1) a merge of locations, reducing the amount of locations where dialogues could be performed, 2) saturation in terms of central themes and new learnings in the dialogues.

### Data collection

Central themes and desired changes of the intervention were defined with stakeholders and continuously monitored throughout the evaluation. Perceived changes of the intervention were evaluated both at fixed moments (i.e. after one and two years) and continuously (Fig. 1).

Semi-structured interviews and participant observations were used to define the central themes and desired changes of the stakeholder dialogues according to the stakeholders (Fig. 1, baseline), and to evaluate the perceived changes during and after the stakeholder dialogues (Fig. 1, evaluation 1 and 2). Topics of the interviews at baseline and at evaluation moments are described in the Study protocol [13]. Periodic Medical Examinations (PME) were used as an additional source of data to verify the scope of the central themes throughout the organization. In addition, all forms of communication with stakeholders (e-mails, logs of phone calls) served as an additional source of data for evaluation.

### Data analysis

Thematic content analysis was performed to analyze data from interviews and dialogues. Analysis about the relevant themes for and desired outcomes of the stakeholder dialogues (baseline) and the perceived changes (evaluation 1 and 2) proceeded inductively. Perceived changes were categorized into changes on four levels, namely case, individual, team, organizational level. These levels were based on the four aims of MCD, the type of stakeholder dialogue used in this study [30], and the EURO-MCD classification [24]. All interviews were first individually coded. Subsequently, comparisons and differences between interviews were made. Atlas.ti 9 Windows was used for qualitative analysis (Coding trees can be found in the Additional file 2). Analysis of the stakeholder dialogues also proceeded inductively, and perceived changes of the dialogues were also categorized into the aforementioned four levels.

### Quality measures

Several quality procedures for qualitative research were taken, as recommended by Frambach et al [31]. These measures are described in the Study Protocol of this study [13] and reflected upon in the discussion.

More details about the methods can be found in the Study Protocol published elsewhere [13].

## Results

The results are presented in two parts, following the research aims. Part I describes the central themes that were defined with the stakeholders. Part II describes the desired changes before the intervention, and the perceived changes during and after the intervention.

### Part 1 – Central themes

Two relevant health related themes stood out throughout the entire evaluation period: high workload and mental health.

#### High workload

This reoccurring theme was often attributed to the unpredictable nature of the work, leading to high peaks and insufficient numbers of personnel. For operational employees, high physical job demands (working with dangerous goods) and mental job demands (multitasking, prioritizing on the spot) also contributed to perceiving a high workload. According to employees, high workload influenced health by disturbing the work-life balance, working less safe, reduced job satisfaction or mental pressure of the potential consequences of mistakes and unsafe working (e.g. losing clients). Working less safe (not fully according to the safety regulations) was especially a concern for younger employees with little experience according to supervisors:

*“Those young boys that just got employed, you have to tell them: dude, calm down. They think: how can I do this as quickly as possible? And then they start running and flying, but you shouldn’t do that. Because with doing that in this job, you risk your safety. They are like oh I forgot to put my helmet because I was too busy.”*—Supervisor, baseline interview 1.

The consequences of mistakes, i.e. not following the safety rules or other mistakes because of a perceived high workload, could be far-reaching. Employees seemed to have a feeling of responsibility regarding the reputation of the organization.

*“We are talking about cargos of over hundred million sometimes. If you make a mistake because you are mentally out of the world for a moment, yes then…” … “If something happens at our plant, [name organization] will take the blame.” –* Operational employee, baseline interview 2.

#### Mental health

Employees and management noticed an increase of colleagues that were absent because of a burn-out or stress symptoms. Periodic Medical Evaluations (PME) that were performed during the course of the project (June 2018 & April 2019), showed that employees with a low SEP scored below national averages on aspects of mental health such as work engagement and above on burn-out and stress. Masculine norms were reported as a contributing factor to burn-out. Keeping the image of being a strong worker and preferable not showing vulnerability impeded employees to speak up at an early stage, even though it was mentioned that the organization is helpful when someone has mental complaints,

*“They are, after all, a bit young guys, uh yes how do you say that politely? Hard working people, you see? It is really what you see in the news, the Rotterdam mentality.”* – Supervisor, baseline interview 1.

*“We are here with kind of tough men and it’s not cool of course to say, yes, things are not great at home or I don’t feel so good.”… “Usually we see it when it’s too late. You notice that people are mentally absent, and then all of a sudden they have a burn-out.”* – Operational employee – baseline interview 2.

#### Topics for moral case deliberation

Based on the overarching themes high workload and mental health, topics for the dialogues were formulated. The researchers searched for concrete examples of the formulated central themes in the data. The topics were discussed with the contact person of the organization. Table 1 presents an overview of the topics and dilemmas of each session is presented.

**Table 1:** Topics and dilemmas in the dialogue sessions

Session	Topic	Dilemma discussed (brought in by participants)
1	Balance between working fast and safe	Being a good employee and colleague *or* working safe and healthy
2		Protecting reputation *or* protecting health
3		Being a good employee and colleague *or* working safe and healthy
4	Discussing (health and safety) issues with colleagues and supervisors	Speaking up *or* being a good employee and colleague
5		Speaking up *or* being a good employee and colleague
6		Own responsibility *or* strict regulations
7	Discussing burn-out with colleagues and supervisors	Help a colleague with burn-out symptoms or protect his reputation
8		Protecting own reputation *or* receive support

### Part 2 – Perceived changes

#### Desired changes before intervention

Stakeholders were asked what they considered relevant changes of the intervention [20]. Interviewees were interested in learnings, either non-specified (i.e. cross pollination about how other departments deal with problems), or more specified (e.g. about how employees in other departments experienced the high workload). In addition, employees from various departments indicated that the dialogues could help defining shared experiences and/or structural issues that require improvement. The dialogues could be a means to jointly come up with ideas for improvement for the decision makers, thereby creating bottom-up support:

“*I mean, if everyone says the same thing.. then the organization has something to work on.”* – Operational employee, baseline interview 6.

The management team was also interested in learnings for improvement. For example, they indicated that it was relevant for them to learn how to could communicate more effectively with the ‘shop-floor’.

#### Perceived relevant changes after intervention

Changes were perceived on all four levels (case, individual, team, organizational). Table 2 presents an overview of all perceived changes with a thick description of the context showing the relevance of the changes for the stakeholders.

**Table 2:** Perceived changes of the dialogues [ MCD = Moral Case Deliberation]

Level	Outcome	Exemplifying quotes context
**Case**	Awareness of work pressure High workload was an issue of concern in the organization, that was reported from the start of the evaluation. However, during the course of the evaluation there were moments that the workload increased even more, among other things because a competitor started to hire experienced employees from the organization under study When this workload was discussed in one of the dialogues, it remained an issue of concern for a while afterwards. Attention was paid to work pressure in the form of toolbox sessions. Toolboxes did already take place every month but some were now especially organized to help employees to cope with the high workload. Some interviewees were, however, critical on the content of the toolboxes	*“We see that people just resign due to work pressure.”* Operational employee – MCD-session 4 *“We did pay some attention to it in one of the toolboxes, work pressure and how you deal with it and what the symptoms are, etcetera. The message was: tell it to your manager, talk about it and don’t surpress it.”* Safety manager – First year evaluation interview *“They say: ‘you shouldn’t feel pressure’. But look, if two men are missing, you have to walk a little faster. If you start working at a normal pace, you leave the work for the next shift, next shift, and it just keeps piling up.”* Operational employee – First year evaluation interview
**Individual**	Give voice Various dialogues showed examples of misunderstandings between management and the operational employees. Overall, the management wanted the operational employees to be more proactive, whereas the employees said that the management was unreachable for them when they wanted to raise their concerns about something The dialogues were a way for operational employees to raise their concerns with regard to certain health issues to the management, under the condition that a summary of the main points discussed was given to the management team	*‘Well, I finally managed to get raincoats after I’ve been asking for them for one year.”* Operational employee—MCD-session 1 *“You hardly see the management. I don’t think they really know what it’s like out here in our world.”* Operational employees—MCD-session 5 *“I think it’s good that they (the management) eventually hear what is discussed in this dialogues. If it doesn’t reach them, then why did we do it?”* Operational employee—MCD-session 6
	Recognition and learnings Employees from various departments reported that they their workloads were too high. In some case this had already led to burn-out. The—mostly male—employees were reluctant in asking for help when they felt they could not handle the workload anymore, partly to keep up their reputation in the masculine environment At the end of the dialogues, participants said that they had realized that everyone, not only them, was sometimes struggling with high workloads. Through this recognition, some participants realized that asking help, for example in prioritizing work, is a valid thing to do in times of high workload	*‘Well, it’s all go, go, go, run. You’re a man and go.”* Supervisor—MCD-session 7 *There are plenty of people here that are afraid to cross that threshold (to speak up/ask help). And if you (as a supervisor) just say: ‘you know, it’s no problem’, it will be easier for them.”* Supervisor – MCD-session 7 *“I think perhaps after this, after such conversation, one can think ‘Oh, I cán discuss things. If something bothers me I can say to my colleague or my immediate supervisor: well, I am with”…”I need help with something.”* Support staff employee—MCD-session 2
**Team**	Enhanced mutual understanding The different departments in the organization were highly interdependent to perform their core tasks. However, the dialogues revealed that employees from the various departments were putting pressure on each other to work faster, which contributed to the high workload Participants of the dialogues said that the dialogues helped to better understand the employees from other departments. They indicated to have realized that employees from other departments also experience high workloads and therefore put pressure on the others. Because of this understanding, they said to have the intention to approach each other more gently	*“Being called every time, that just doesn’t work”…”If you tell the [employee from other department], dude, one request take me two hours, and you expect me to do two in three hours..”* Operational employee—MCD-session 5 *“I don’t know exactly what happens in the other departments. I don’t know all that. But now you hear everything a bit and then you can also empathize a bit them, like, be considerate about these people.”* Operational employee – MCD-session 4 “*You would say that some departments are less busy, but no. They all experience that high pressure. That does make you wonder.”* Supervisor – First year evaluation interview
	Shared insights The little consultation moments were brought forward as an issue of concern in several dialogues. Participants said that there were few moments of consultation with colleagues and supervisors. Especially in times of high workload this moments were scarce, while such consultation moments were considered important to give the operational employees the chance to raise their concerns The management took initiatives to promote consultation moments (also see organizational level changes)	*“I’ve already told the management, go sit down with the shifts every six weeks”…”That you all get the feeling again, guys, we are going that way.”* Support employee—MCD-session 5 *“People miss information. That’s what I take from this conversation. And that it is very important that people feel heard.”* Safety manager – MCD-session 5 *“You just realize that sometimes the employees could use a listening ear a little more frequently”…”And that it would be good if that takes place more often, so that people can vent a little more.”* Safety manager – First year evaluation interview
**Organizational**	Organizational learning process The management indicated to have gotten a better view of the daily experiences of operational employees. For example when it comes to the doubts of employees with little experience when judging the safety of a situation. Understanding this, encouraged the management for example to emphasize to the employees that can use a symbolic card to stop working temporarily when in doubt of the safety of the situation Also, signals from employees seemed to be taken more seriously. For example with regard to the high workload employees brought forward as an issue. At first, the management could not match this with the number of orders from clients. But by listening more to the stories of employees, they found out what factors did actually contribute to this workload, apart from the number of client requests	*[“Researcher: what were insights you gained during this study?”]…”Well, discussing things like, even though we emphasize constantly on things like [the symbolic card to stop working temporarily when doubting about the safety of a situation], that in practice we understand that this is different than when you just write it down formally.”* Member of management team – Second year evaluation interview *“You see in request from clients that compared to last year that it is a lot less busy, while that is not the feeling with people, they experience pressure. And then during the toolbox sessions we’ve discussed this a few times and I realized it is not in the number of requests, but more due to people being ill or on leave.”* Safety manager – First year evaluation interview
	Implementation of a program to enhance mutual understanding Based on the feedback that the management received from the dialogues about the tensions between departments, it decided that all employees (including management) were required to spend one day with an employee from another department. The aim was to get a better understanding of each other’s work processes, which should contribute to better collaboration between departments and to soothe tensions between management and operational employees	*“It starts at the customers. They are business-minded and they only think about the money, and that mentality is transfered to the management here”…”Which makes account managers think like that, who pass it on to the shop floor. That’s kind of the problem. We are always, the shopfloor, at the bottom of the chain.”* Operational employee—MCD-session 1 *“The program Visible Leadership will be reintroduced and an become an objective for all employees. This gives each employee the opportunity to gain more insight in the work of the other, with the intended effect that mutual understanding is created and collaboration improves.”* E-mail from management team to all employees – one year after the start of the project
	Improving internal communication The dialogues revealed that the internal communication was contributing to the high workload in various ways. The participants indicated that the management could for example, better inform the employees about the acquisition of new clients, so that they could prepare themselves better for the upcoming extra work Also, within departments the communication could be improved for the purpose of the safety of employees: topic of several dialogues were situations in which the safety of that situation was hard to judge. Often because of extreme heat, or very cold weather. Although extensive safety guidelines were used to judge the safety of a situation, peculiar situations in the case of extreme heat or cold, required experience to accurately judge the situation. A solution that was brought up in the dialogues was short consultation moment with the supervisor or a colleague in such situation and in case of doubt	*“In recent years it has surprised me sometimes: suddenly a new customer, that I didn’t know about. And then you hear, yes, we brought those in, and I have not seen an email at all. Then we had more work again. It’s just there, all of a sudden.”* Operational employee—MCD-session 4 *“Yes, that one person thinks, oh, it’s safe, I’m going to do it. And the other says, no, it’s unsafe after all. But then they are talking about the same situation.”* Operational employee – MCD-session 3
	Implementation of a program to enhance engagement and appreciation Employees experienced a lack of appreciation from the management. Both interpersonally and financially. Both were related to the fact that the organization is part of an American company, that for a large part out sets out the policies for the organization under study	…”That it becomes more American”…”the whole company, you see it in everthing”…”More distant, it becomes more distant” Supervisor – MCD-session 7 *“But you don’t hear about it from the management. That I find regrettable: let those guys know, thanks guys, I couldn’t have done it without you.”* Supervisor – First year evaluation interview
	More preventative approach on burn-out Burn-out was on the rise in the organization. Supervisors and colleagues said that it was hard to notice symptoms at an early stage. One contributing factor was the masculine culture, in which it was not considered ‘man enough’ to tell your colleagues when you are not well One of the conclusions from the dialogues was that supervisors can pay more attention to the mental well-being of employees. HR urged supervisors to pay attention to signals and to send employees on temporarily leave when they consider that necessary to prevent complete fall out	*“You say, I’m a guy, I work hard, I can handle stress. And after three weeks you leave because you can’t handle the stress while you wanted to grow in the company. Then, of course, people will look at you differently. So you get over it and say, I can do this.”* Supervisor – MCD-session 8 *“Well, I try to urge the supervisors like, you are the first to see or notice something about people. And with quite a few people, before getting absent I’ve had a chat, saying ‘just take a step back’. Do just something different. And all of that has actually resulted in people that were absent for one or two weeks, but after that came back to work.”* HR-manager – Second evaluation interview

Below, one change per level is described in detail. We selected changes that were not a single event, such as the purchase of a safety means, but were assumed to have a longer-term duration (e.g. perceived enhanced mutual understanding).

#### Case level

##### Agenda setting

Some dialogues led to follow-up discussions about topics similar to the ones discussed in the dialogue. These follow up discussions were initiated by the organization, rather than by the researchers. For example, after a dialogue in which the peak of workload at that moment was discussed, ‘toolbox’ sessions were organized about the experience of high workload. In these sessions it was discussed how to prioritize tasks and how to deal psychologically with high workloads.

One operational employee that participated in a dialogue mentioned in the evaluation that the effort to reduce (the experience of) high workloads increased strongly directly after the dialogue. Yet, it was emphasized that this attention decreased after some time when the workload increased again. Nevertheless, changes on other levels occurred that were also related to the experience of high workload.

#### Individual level

##### Recognition and learnings

The dialogues led to recognition of issues for the participants of the dialogues. Participants realized that colleagues, either from the same of from different departments, experienced similar issues, such as the high workload. It was reassuring for participants of various departments to realize that their department was not the only one experiencing high workload, but that it is a companywide issue. Also, the dialogues revealed that the prevailing masculine norms like being a strong worker, preferably not showing vulnerability, prevent employees from asking help. Participants indicated that they realized during the dialogue that asking for help in times of very high workload is a legitimate thing to do. Participants also realized that it may also be helpful for other employees not participating in the dialogues to know that it is not a problem to ask for help and that this should be communicated more actively.

#### Team level

##### Perceived enhanced mutual understanding

Participants mentioned that the sessions contributed to enhancing the mutual understanding between departments. Tensions between departments, that are strongly interdependent for their core activities, was a factor that contributed to the experience of high workloads. Participants of the dialogues indicated that they sometimes got surprised by the perspectives of employees from other departments. Insight in their perspectives and working conditions enhanced understanding for certain situations that contributed to the experience of high workload. Moreover, the organization implemented an exchange program between departments to enhance the mutual understanding further.

#### Organizational level

##### Organizational learning process

The dialogues helped the management to better understand the underlying factors of the central themes, high workload and mental health. From the perspective of the management, there were no signals about an increase in workload; there was no increase in requests from clients. However, during the course of the project, members of the management team started to learn via the dialogues what were the underlying reasons for the perception of high workload. Insight in these reasons, such as the sometimes compelling communication and tensions between departments, allowed the management to take targeted actions. For example, the management implemented a communication training for supervisors to promote respectful communication and proactiveness of employees in order to involve them more in daily practice. Other actions that were taken by the management were the implementation of an exchange program with the aim to learn about each other’s work, initiatives to enhance the engagement of employees in organizational developments and stimulating a more preventative approach on burn-out by making supervisors aware that they are the ones that can signal symptoms at an early stage.

## Discussion

This paper describes the evaluation of an innovative WHP study in which central themes for and desired changes of the intervention were defined together with employees with a low SEP and other stakeholders. High workload and mental health turned out to be wide-spread issues in the organization under study. In the stakeholder dialogues, participants shared examples of their own experiences with these themes. This initiated a learning process in the organization, in which the management gained more understanding of the factors playing a role in mental health and high workload. In reply to this, several actions were implemented on the organizational level.

An unique future of this study was the active role employees played in defining the central themes of the intervention. Participatory research designs are not yet common in the field of WHP, although they have been recommended [11, 32, 33] and explored [34, 35]. In a classification of the degree of participation in participatory research from Fetterman [36], this study could be classified in ‘Collaborative Evaluation’. There was ongoing engagement between researchers and stakeholders. However, the researchers remained in charge of some of the main decisions, such as the method of the intervention, as well as for the methods of evaluation, although they were adjusted to the work setting under study. In the classification of Fetterman the Collaborative Evaluation is the lightest form of participation. Nonetheless, on the ladder of participation of Arnstein [37], this study could be placed on step six ‘Partnership’ (the ladder includes step one to eight, eight being the highest degree of participation).

The stakeholder dialogues are expected to have contributed to health of employees with a low SEP in two ways. First, through the actions that followed from the dialogues. Most of the actions related to improvements in the work context. It has been shown that working conditions contribute as much and sometimes more than healthy behaviors to health of employees with a low SEP [38, 39]. Second, participants of the dialogues reported learnings after participating. A concrete example being the insight that asking for help in busy times can be considered a legitimate thing to do. Employees may have profited from this learning in situations in which they had high workloads.

Next to the actions, the group composition in the dialogues—mostly homogeneous groups in the sense of dependency relations—may have been advantageous to employees and social relations in the organization. Although one of the reasons to study a stakeholder dialogue as an intervention was the variety of stakeholders involved in WHP, mainly one stakeholder group participated in the dialogues, namely employees, although from different departments and with a variety of functions, aligned with their preferences [13]. Homogeneous groups may be advantageous in hierarchical organizations – such as the organization under study—because they allow for so-called ‘enclave deliberation’, in which like-minded people discuss topics together. This has been shown to enhance self-efficacy and interpersonal trust [40] and might as well have established a safe communication climate [25]. It may also help to deal with power differences between groups and forestalls domination by established groups [41]. However, which group composition is favorable depends on the power relations in the organization where the intervention is implemented.

It should be recognized that there were several favorable circumstances for Responsive Evaluation and stakeholder dialogue. First, the organization under study allowed that the dialogues took place during working time. This probably enhanced the willingness of employees to participate. Second, the organization was open for feedback, a requisite for participatory research to succeed [42]. Possibly, this openness was related to organization’s focus on safety and the associated continuous attention for improvement. However, the first dialogue yielded a lot of response. Some participants expressed their frustration about other participants who, in their eyes, used the dialogues as a platform to ‘just’ express their frustrations without being constructive. The turmoil evoked worries about the upcoming dialogues, also at the higher level management. In the following dialogues, the researchers paid more attention to the underlying concerns of the expressed frustrations and on what could be helpful to these concerns. Similar strong responses on the dialogues did not occur again. In fact, the strong reactions on the first dialogue were in hindsight perceived as a sign that employees should be heard more regularly.

Also, the gender of the researchers (both women) may have played a role in how health issues were discussed in the dialogues. The researchers noticed that participants were spoke openly about issues such as mental health and high workload in dialogues and interviews, while the same participants mentioned that there was a lack of openness about these issues because of the prevailing masculine norms. Possibly, the participants felt comfortable about discussing the themes because they perceived the female researchers as ‘empathic listeners’ [43], and being women, ‘allowed’ to care and ask questions about health [44]. Also, the researchers paid explicit attention to their language. They based their language on how employees themselves talked about mental health and high workload in participant observations and interviews. For example, participants never used the word ‘stress’, but used ‘high workload’. This may have contributed to a safe communication climate.

### Strengths and limitations

A strength of this study was the variety of data sources (data triangulation [31]) used to identify and monitor the central themes. The combination of interviews, participatory observations, PME-data and the dialogues allowed to get an varied view of the issues and the factors related to it. Also, the interpretation of the results took place in consultation with the participants (member check (30)). After each interview and group dialogue, the participants received a short summary made by the researchers. Participants could adapt or approve these summaries, thereby serving as a member check to verify the correctness of the interpretations of the researchers. After approval of the participants, the summaries were used to inform the higher management about the dialogues. This feedback loop was strongly valued by the participants; without informing the decision makers there would not be no further impact on their daily working life.

The type of evidence provided with this study can be considered a limitation. The perceived changes were identified by means of qualitative data. No statistical evidence was gathered about the effects of the intervention This impeded comparison of findings of various studies in a statistical manner. Fortunately, the qualitative data were informative on the experiences and perceived changes of the dialogues. The qualitative findings can only be transferred to similar settings (male-dominated large organizations (> 250 employees)), through the ‘thick description’ of the work setting given in the results [42]. The thick description of the work context, stakeholders and circumstances, allows other researchers or professionals to relate the findings to the context of their interest. Another limitation is that the initiated actions on an organizational level, only started to take place after one year. Therefore, it was not evaluated how employees appreciated and were affected by these actions on the longer term.

### Implications for practice and research

Employers can learn from this study that actively asking employees to share health related issues from their daily experience can lead to shared insights about the factors contributing and withholding to their health. New interventions can take from this study that regarding employees as partners in WHP allows to understand the health issues relevant to their daily reality. Through this understanding WHP can be better adapted to the lifeworld of employees with a low SEP.

## Conclusion

The Responsive Evaluation and stakeholder dialogue initiated and facilitated a learning process in an organization around central health themes, high workload and mental health. Although the perceived changes identified in this study are specific for the context under study, other organizations can learn what the result of dialogue with employees can be for their own WHP. Researchers, intervention providers and other stakeholders can take from this study that employees with a low SEP can be reached in WHP by involving them in in the intervention and evaluation. Also, it allows to understand the health issues that are relevant for employees, thereby making WHP more suitable for employees with a low SEP.

## Supplementary Information

**Additional file 1.** Guideline stakeholder dialogue

**Additional file 2.** Coding schemes

## Data Availability

Not applicable.

## Abbreviations

SEP: Socioeconomic position
WHP: Workplace health promotion
MCD: Moral Case Deliberation
