# Supplementary material for: Stakeholder dialogue on dilemmas at work as a workplace health promotion intervention including employees with a low SEP: a Responsive Evaluation
Source: BMC Public Health. 2022 Feb 28;22:407. doi: 10.1186/s12889-022-12802-z (PMC8883621; doi:10.1186/s12889-022-12802-z)
Supplement: Supplementary file 1 — Additional file 1. Guideline stakeholder dialogue [file 12889_2022_12802_MOESM1_ESM.docx]

**Additional file 1 - Guideline stakeholder dialogue***Extensive description of dialogue guideline, with aims and actions
This guideline is based on the ‘Dilemma-method’, a form of Moral Case Deliberation* [1, 2]
 **Adaptations made beforehand**

- **Duration:** we used a shortened version of the Dilemma-method, as described in ‘Handleiding Moreel Beraad’ [3]. We selected this version because of the time that was available for the dialogues in the organization (one hour), while at least one hour and a half is recommended for the full version of the Dilemma-method.
- **Language:** we have adapted the (Dutch version) of the steps of the Dilemma-method to B1 literacy level as much as possible. Online language level tools were used for this purpose. Abstract terms were avoided or explained. For example, a dilemma was explained as: a situation in which you doubt between actions that can both have a negative consequence. Another example of a language adaptation was, instead of asking ‘What is the moral damage of option A’?, we asked ‘What is the disadvantage of option A?’.
- Adaptations made during the evaluation are highlighted in red in the description of the guideline below.

Practicalities before dialogue:

- Bring/organize flip-chart to summarize answers participants, as indicated at each step. Various pages of the flip-chart will be needed.
- Bring informed consent forms, pens and an audio-recorder.
- Send an invitation via e-mail a few days before the dialogue to the participants. The e-mail should inform participants about the theme of the dialogue and contain instructions to think about a dilemma with regard to the theme before the meeting.
- **Added during evaluation:** Bring an image related to the theme of the dialogue and place it at a visible place in the room. *Explanation adaptation: images turned out to evoke more associations with the theme than a verbal explanation alone.*

**Introduction***Background:*

1. Introduction of moderator and present researchers.
2. Explanation of research and research activities so far.
3. Explanation of the theme of the dialogue and check its relevance for the participants in the dialogue.

*Aim of the dialogue:*

1. Explanation of the aim of the dialogue
   - learning from each other;
   - sharing experiences/issues at work with regard to health;
   - learn how to deal with/ finding solutions for these issues.

*Before the dialogue starts:*

1. Short introduction of all participants.
2. All participants share their dilemmas/situations (some participants may already have formulated a dilemma, for others the dilemma has to be found in consultation with the moderator and other participants).
    *Write key words of all dilemmas/situations on the flip-chart.*
3. Moderator repeats situations and asks participants to vote for one the situations.

**Step 1: Defining the situation**After deliberately selecting one situation for the dialogue, the situation has to be understood well by all participants in the dialogue.

*Aims of this step:* define dilemma, all participants understand the situation that was brought in, formulate two concrete actions in the situation, and formulate the negative consequences of both options.

1. *To person whose situation is chosen*: Please explain the situation in a bit more detail.

   *Probing questions:* where were you? What were you doing? What was it that you were doubting about? How did you feel about it? Which concrete actions can be attached to your doubt?

*Write key words on the flip-chart.*

1. *To all participants:* Do you still miss information to understand this situation?

*Probing questions:* when did this happen? Why? To what extent? What does X mean? How did that look like? From where came..? What were causes?

1. *To person whose situation is chosen:* What could you do in this situation? Name 2 options.

*Write option A and option B on the flip-chart.*

1. *To all participants:* What could be disadvantages of option A? And of option B?

*Probing questions:* why is that an disadvantage? What could happen if you make that decision?

*Write disadvantages of choosing option A and B on the flip-chart.*

**Step 2: Consequences and stakeholders***Aims of this step:* understand what would motivate participants to go for option A, or B, which values play a role, which other parties are involved in this situation, and understand what other stakeholders/parties find important in this situation according to the participants in the dialogue.

1. *To all participants:* Why would you select option A? (What is the advantage of A), and for B?

*Probing questions:* why would you do that? What motivates you there? Why is that important for you? What will you achieve with that? What makes that you say that?

*Write motivations of choosing option A and option B on the flip-chart.*

1. *To all participants:* Which other stakeholders/parties are involved in this situation?

*Probing questions:* who’s affected by your decision in this situation? Who has a stake in this situation?

*Write involved stakeholders/parties on the flip-chart.*

1. *To all participants:* What is important for these stakeholders/parties?

*Probing questions:* What do you think they would want? Why? What is their objective? What makes you think that? What is their interest?

*Write interests of stakeholders/parties on the flip-chart in keywords, if possible translated to values.*

1. *To all participants:* What other options do you have in this situation, apart from A and B?

*Probing questions:* are there alternatives to option A and B?

**Step 4: Conclusion***Aims of this step:* understand what participants would do in this situation, understand differences and similarities in the decisions for A or B or alternatives, understand how negative consequences of the options could be minimized, and understand the practical actions/measures on an organizational, team and/or individual level needed for this.

1. *To all participants:* What would you do in this situation? Would you go for option A, B, or an alternative? Why? (ask person whose situation was brought in first).

   **Adaptation made after first three dialogues:** *In the first three dialogues participants were asked to fill in a form. On the form they were asked to write down what option the participant would choose, why, how the negative consequences of that option could be avoided and what would be practically necessary to go for this option. Participants indicated that they did not understand what to write and that they did not like it. They preferred to explain their decisions verbally. Therefore, we decided to ask the questions that were on the form verbally.*

*Write down the different answers on the flip-chart. Write down count if possible.*

1. *To all participants:* What are similarities in your answers? What are differences in the answers?

*Probing questions:* why did some choose option A/B? The majority selected A/B, why did you choose differently? Can you explain? What can we learn from this?

1. *To all participants:* How can we diminish the negative consequences of the option you’ve selected?

*Probing questions:* looking back at the disadvantages formulated for this option, what can be done to minimize those? What would you need for that? Whose support do you need for this?

*Write down concrete actions/solutions that minimize the negative consequences of the options on the flip-chart.*

1. What do you practically need for your decision?

   *Probing questions:* who is responsible for that? Who should be involved? What is a next step?

*Write down practical needs on the flip-chart.*

**Step 5: Rounding up***Aim of this step: formulate concrete actions and objectives.*

1. *To all participants:* Which concrete agreements can we make now?

*Probing questions:* what could you do now, on short-term? What would you need from others, organization, team, supervisor?

*Write actions and objectives on the flip-chart.*

**Step 6: Evaluation***Aim of this step: evaluate the experiences with the dialogue.*

1. *To all participants:* What did you think of this dialogue?

   *Probing questions:* How did you experience being in a dialogue? Was it difficult, easy? Did you hear anything from others that you will take with you?

**End of dialogue**

1. *Conclusion by moderator:* explain what will be done with the formulated actions:

- A short report will be made; participants can read this report and make adjustments if deemed necessary.
- The short report will be shared with management.
- Planning of coming dialogues.
- Questions?

[1] Molewijk AC, Abma T, Stolper M, et al. Teaching ethics in the clinic. The theory and practice of moral case deliberation. *J Med Ethics* 2008; 34: 120–124.

[2] Van Dartel, Hans., Molewijk B. *In gesprek blijven over goede zorg - overlegmethoden voor moreel beraad*. Uitgeverij Boom, Amsterdam 2014, 2014.

[3] de Bree M, Veening E. *Handleiding Moreel Beraad*. 2nd ed. Koninklijke van Gorcum, 2016.
