# Supplementary material for: Stakeholder dialogue on dilemmas at work as a workplace health promotion intervention including employees with a low SEP: a Responsive Evaluation
Source: BMC Public Health. 2022 Feb 28;22:407. doi: 10.1186/s12889-022-12802-z (PMC8883621; doi:10.1186/s12889-022-12802-z)
Supplement: Supplementary file 2 — Additional file 2. Coding schemes [file 12889_2022_12802_MOESM2_ESM.docx]

**Additional file 2 – Coding schemes**


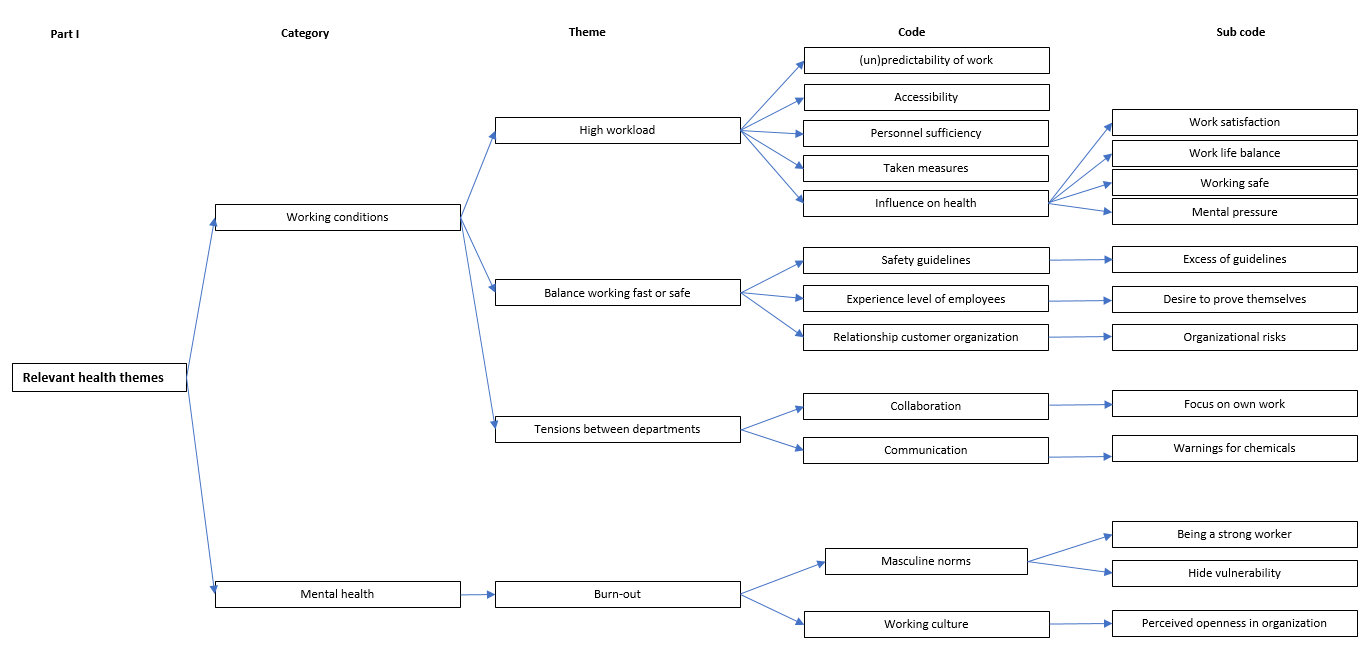


**Figure 1.** Coding scheme 1. Part I: health themes


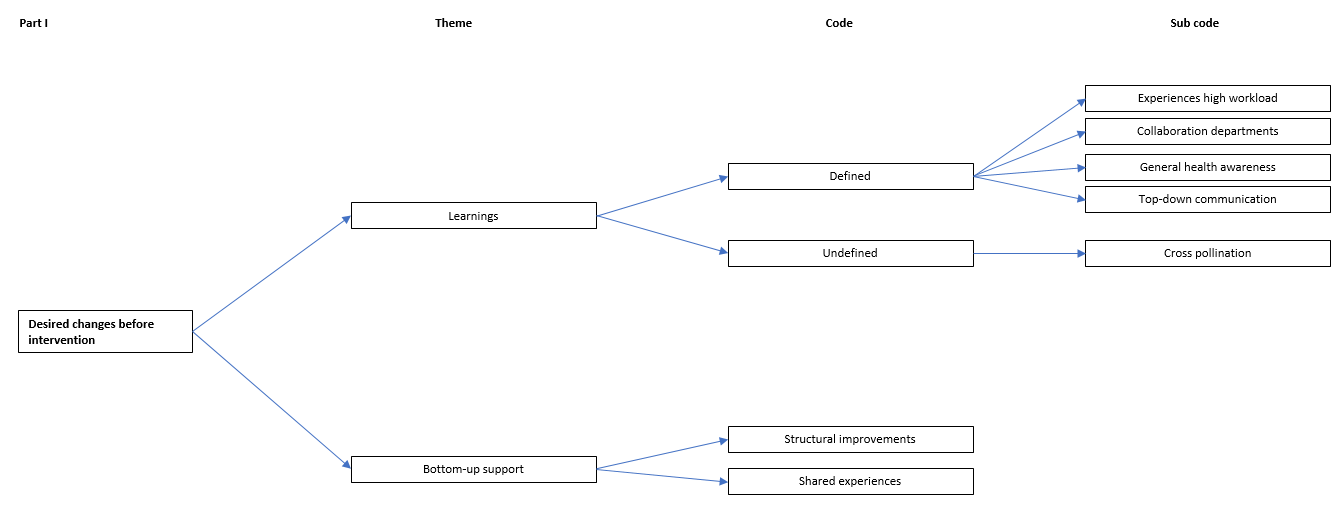


**Figure 2.** Coding scheme 2. Part I: desired changes before intervention


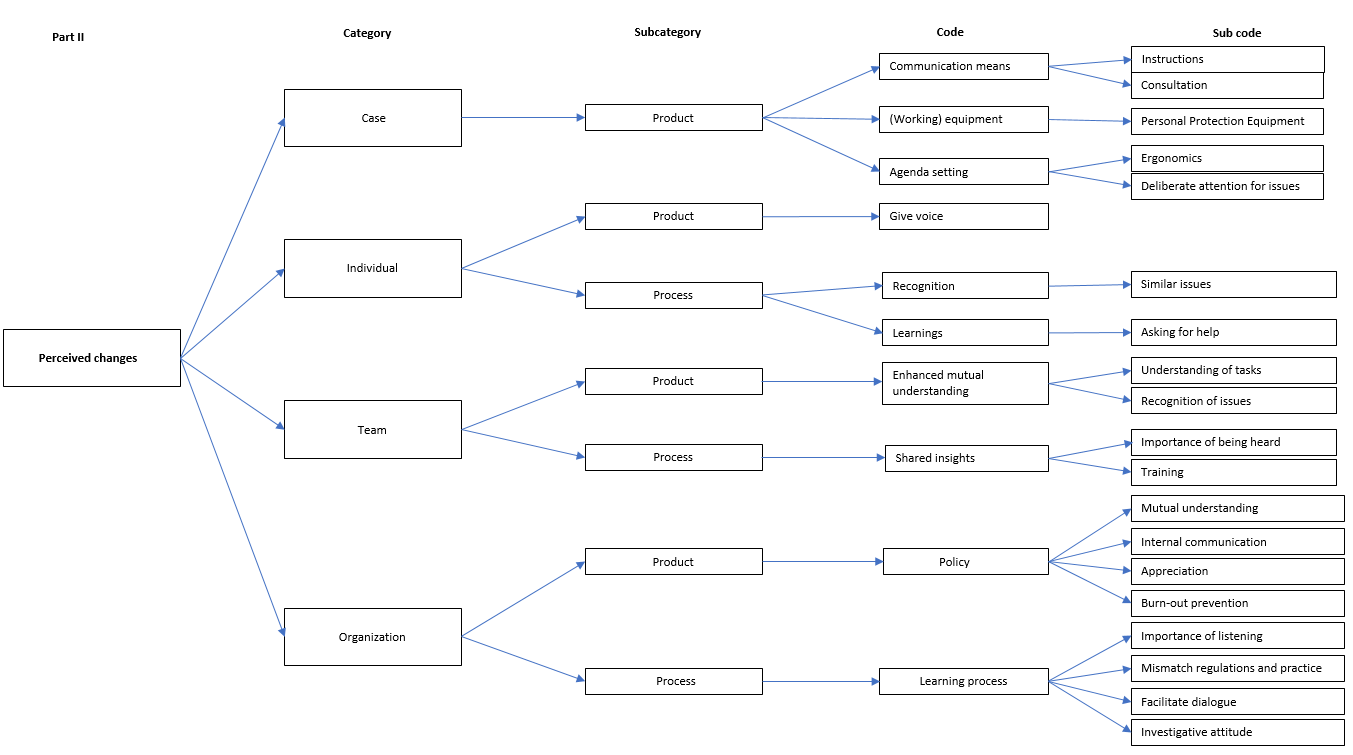


**Figure 3.** Coding scheme 3. Part II: perceived changes after and during the intervention


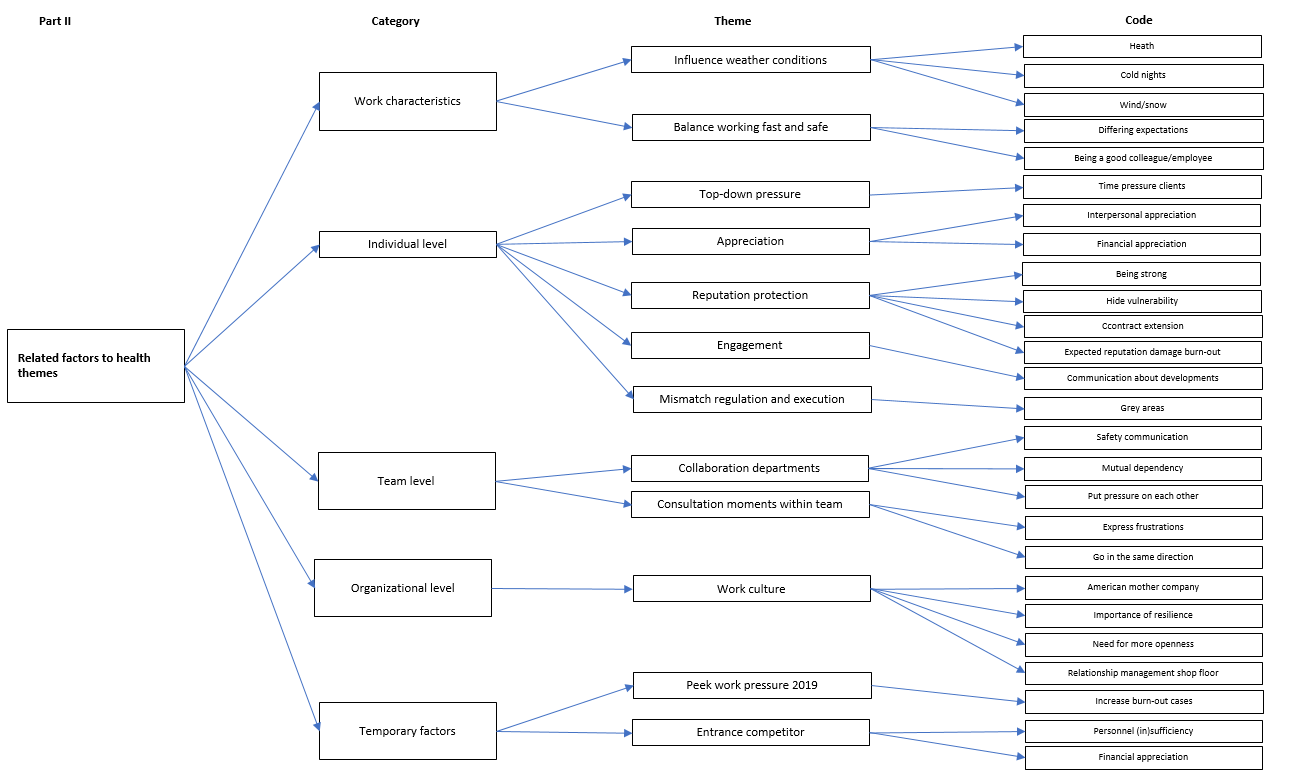


**Figure 3.** Coding scheme 4. Part II: Factors related to health themes
